# Molecular Approaches to Identify Cryptic Species and Polymorphic Species within a Complex Community of Fig Wasps

**DOI:** 10.1371/journal.pone.0015067

**Published:** 2010-11-29

**Authors:** Jin-Hua Xiao, Ning-Xin Wang, Yan-Wei Li, Robert W. Murphy, Dong-Guang Wan, Li-Ming Niu, Hao-Yuan Hu, Yue-Guan Fu, Da-Wei Huang

**Affiliations:** 1 Key Laboratory of Zoological Systematics and Evolution, Institute of Zoology, Chinese Academy of Sciences, Beijing, China; 2 College of Plant Protection, Shandong Agricultural University, Tai'an, Shandong, China; 3 State Key Laboratory of Genetic Resources and Evolution, Kunming Institute of Zoology, Chinese Academy of Sciences, Kunming, China; 4 Department of Natural History, Royal Ontario Museum, Toronto, Ontario, Canada; 5 Environment and Plant Protection Institute, Chinese Academy of Tropical Agricultural Sciences, Danzhou, Hainan, China; VU University, The Netherlands

## Abstract

Cryptic and polymorphic species can complicate traditional taxonomic research and both of these concerns are common in fig wasp communities. Species identification is very difficult, despite great effort and the ecological importance of fig wasps. Herein, we try to identify all chalcidoid wasp species hosted by one species of fig, using both morphological and molecular methods. We compare the efficiency of four different DNA regions and find that ITS2 is highly effective for species identification, while mitochondrial COI and Cytb regions appear less reliable, possibly due to the interference signals from either nuclear copies of mtDNA, i.e. NUMTs, or the effects of *Wolbachia* infections. The analyses suggest that combining multiple markers is the best choice for inferring species identifications as any one marker may be unsuitable in a given case.

## Introduction

The accurate identification of an organism—taxonomy—forms the cornerstone of most aspects of biological science. In most groups, traditional taxonomic research is based on morphological characters. This can result in many, sometimes intractable, problems, especially in groups that have both cryptic and polymorphic species. Cryptic species, two or more distinct species that are erroneously classified (and hidden) under one species name, have challenged taxonomic research for nearly 300 years since they were firstly recognized, and even before the Linnaean classification system was adopted [1], [2]. In contrast, a polymorphic species can have spatial and temporal heterogeneity. Many morphological differences can occur within a species and this can express itself, for example, as extreme sexual dimorphism, larval vs. adult morphology, epigenetic development, and geographic variation. In traditional taxonomy, the problem can be acute when either one or the other sex is rare or difficult to collect [3].

Double trouble occurs when both cryptic and polymorphic species co-occur within a species complex, as happens in fig wasps. The biological and morphological characteristics of fig wasps are very complicated due to their long history of co-evolution within the peculiar, closed environment of fig syconia [4], [5].

Though difficult, the accurate identification of fig wasp species is crucial for further study of these diverse and ecologically important species. Fig wasp communities exhibit much variation in composition, behavior, and ecological and biological diversification [6]. Fig wasps also have many biological characteristics that predispose them to serve as model organisms in studies of adaptation, sympatric speciation, and female bias sex-ratio strategies, among other topics [7], [8], [9]. The conclusions of such studies stand or fall with correct taxonomy. Assessments of ecological neutral theory [10] also require an accurate taxonomy to successfully put groups of species into their correct ecological context.

Traditionally, fig wasp species have been identified by their morphology, but this is often difficult and error-prone. Extreme wingless polymorphic males are found in many species, but are often impossible to correctly associate with conspecific females, because mating has not been observed [11]. In addition, many species have multiple male morphs that can differ greatly from each other. Because morphological approaches often fail, fig wasps are an excellent model system for testing the efficacy and accuracy of species identification using molecular methods.

DNA barcoding strives to identify all species using nucleotide sequences from a homologous fragment of one mitochondrial DNA gene, cytochrome oxidase subunit 1 (COI). This new approach can be both fast and cost effective, and the data are easily standardized and compared across taxa. Although most published studies document success with the standard barcoding approach, there are some general theoretical issues. For example, COI is maternally inherited and its patterns of diversity and evolution can be influenced strongly by inherited microorganisms, such as *Wolbachia*. In addition, there can sometimes be technical and analytical problems due to NUMTs (mitochondrial genes integrated into the nuclear genomes) [12], [13].

Several molecular phylogenetic studies have focused on fig wasps and have used a variety of different molecular markers, such as COI, COII, Cytb, 12S, 28S and ITS2 [14], [15], [16], [17], [18], [19], [20], [21], [22]. Most of these studies focused on species that pollinate fig flowers and no study has attempted to identify all the species in a fig wasp community hosted by one species of fig. In some cases, these communities can involve more than 30 morphospecies with unknown further cryptic diversity. Further, the utility of the different markers adopted in fig wasp molecular studies has not been systematically compared.

Herein, we investigate the species diversity of chalcidoid wasps within the small compact world of the syconium of *Ficus benjamina* (Moraceae). We first identify the morphospecies based on the characteristics of females and then add molecular evidence from both the nuclear and mitochondrial genomes. When the molecular identification results match the morphospecies very well, we consider these consistent results to delimit fig wasp species. The utility of four different molecular markers—EF1-α, COI, Cytb, and ITS2—is evaluated with respect to their ability to identify species in fig wasp communities.

## Materials and Methods

### Taxa sampling and morphological study

From January of 2006 to October of 2009, we surveyed species richness of fig wasps on *Ficus benjamina* (Moraceae) in Hainan province, China. Ripe figs from this monoecious tree, which is naturally distributed mainly in Southeast Asia, were collected in the field and dissected in laboratory. All encountered fig wasps were stored in 95% ethanol. Female specimens were identified to morphospecies and males were provisionally assigned to species (labeled starting with letter ‘M’) by using a Nikon SMZ80 microscope. The dramatic sexual dimorphism and common occurrence of male polymorphism within species dictates this separate approach for male and females Images of the wasps were captured by using a Nikon AZ100 microscope system. Males and females of a pteromalid species, *Dibrachys* sp., collected in Inner Mongolia, China, were also included because many fig-associated genera have been repeatedly moved into or out of this family. Inclusion of this pteromalid, which is not associated with figs, was expected to help clarify the taxonomic positions of fig wasps.

### DNA extraction, PCR amplification, and sequencing

For molecular analyses, DNA was extracted from each specimen and four gene fragments, ITS2, EF1, Cytb and COI, were PCR amplified with conserved insect primers following standard protocols [23], [24], [25]. It is important to stress that the COI fragment amplified here is shorter than the full standard barcoding fragment, but the latter is difficult to amplify and often generate pseudogene sequences in fig wasps. For some individuals, two copies of fragment EF1-α were amplified, and we chose the smaller one corresponding to the F1 copy of *Apis mellifera* [26]. Purified DNA fragments were cloned into Peasy-T1 vector according to the manufacturer's protocols (*TransGen Biotech*, Beijing, China). For each fragment, 1–3 positive clones were sequenced by Invitrogen Sequencing Center, Shanghai. Genomic DNA vouchers and specimen vouchers are deposited in the Institute of Zoology, Chinese Academy of Sciences, Beijing, China.

### Sequences alignment and phylogenetic analysis

Sequences were initially aligned by using ClustalW with default multiple alignment parameters (gap opening penalty = 15, gap extension penalty = 6.66, delay divergent sequences = 30%) for Cytb, COI and EF1-α, and with different parameter settings for ITS2 (gap opening penalty = 10, gap extension penalty = 5, delay divergent sequences = 30%), because this non-coding region evolves with a high incidence of indel events.

Overall similarity of sequences was summarized as Neighbor-Joining (NJ) phenograms obtained using Mega 4.1 [27]. NJ trees for COI and Cytb sequences assumed the Tamura-Nei substitution model, with different rates among sites (α = 0.8). The Kimura 2-parameter substitution model with different rates among sites (α = 0.8) was used for EF1-α. Finally, we used the Kimura 2-parameter substitution model assuming uniform rates among sites for ITS2; gaps and missing data were deleted for pairwise calculations. Bootstrap support values were generated using 1000 pseudoreplicates.

Bayesian inference was employed to estimate phylogenetic relationships using MrBayes 3.12 [28]. Selection of the appropriate model of evolution was selected by using the hierarchical likelihood ratio test (hLRT) [29], which was implemented in the program of Modeltest 3.7 [30]. Calculations used 1 million generations while sampling a tree every 100 generations. A 50% majority rule consensus tree was calculated from the sampled trees. To avoid getting stuck on local optima, we monitored the fluctuating value of the log likelihood ratios graphically and compared the results to apparent stationary levels for at least two independent analyses that started with different seed trees [31]. The first 50% of samples were excluded as “burn-in”. Nodes resolved ≥95% of the time were considered to be significantly supported.

## Results

### Morphological studies

Based on females, we identify 12 morphological species, and examination of the males reveals 24 provisional morphs. All species are illustrated in supplemental Figure S1 and their diagnoses are listed in supplemental Table S1.

Among the 12 chalcidoid species, seven (58%) exhibit extraordinary sexual dimorphism (ESD) and six (50%) have polymorphic males (PM), of which three have more than three morphs. Two species showing PM (*Philotrypesis* sp.1 and *Sycobia* sp.2) have both winged and wingless males. ESD and PM are highly correlated; only two out of seven species showing ESD do not also show PM (*Philotrypesis* sp.5 and *Sycoscapter* sp. 1). In the most extreme example of PM, *Philotrypesis* sp.1 has one winged and five wingless male morphs (Table S4). The latter differ in head shape and size, fore coxae with or without hairs inter-ventrally, head with or without a band of bristles on sides, head with or without a bush of bristles (Figure S1).

### Characteristics of the obtained sequences

The base frequencies of Cytb and COI show a high adenine (A) and thymine (T) bias (Cytb, A+T = 77%; COI, A+T = 74%), which is typical of hymenopteran mitochondrial sequences. In contrast, the base compositions for EF1-α (A = 0.24, T = 0.25, C = 0.27, G = 0.23) and ITS2 (A = 0.26, T = 0.22, C = 0.27, G = 0.25) are much more equal. Among all the taxa, pairwise sequence divergence of Cytb varies from 0 to 28.2%, COI from 0 to 23.1%, and EF1-α from 0 to 18.6%. However, ITS2 shows a much higher level of divergence, from 0 to 83.2%, due mainly to the considerable length variation in this fragment.

Unambiguous alignments are obtained for Cytb, COI and EF1-α. The lengths of ITS2 vary owing to indels. However, this variation is not a problem here, because we are interested only in identifying species. All sequences were submitted to GenBank under the accession number of FJ438013 to FJ438369. The complete alignments used in the final analyses will be provided upon request.

We obtain 89 Cytb, COI and EF1-α gene sequences and 90 ITS2 sequences. The trimmed sequences lengths are 433 bp, 433 bp, and 367 bp for Cytb, COI and EF1-α, respectively. Although the lengths of ITS2 vary from 333 bp to 640 bp among species, the lengths are similar or identical within species. The five sequences of ITS2 from *Philotrypesis* sp.4 have either 402 or 404 bp. Among the 11 individuals of *Philotrypesis* sp.1, all are 377 bp long except for one male morph of 374 bp. All four individuals of *Philotrypesis* sp.5 have identical sequences of 378 bp. Although nucleotide divergence is high between *Philotrypesis* sp.1 and *Philotrypesis* sp.5, sequence lengths vary by only one to four bp. Thus, most individuals are easily associated with the expected species by visual inspection of the aligned sequences.

### Sequence discrimination of species by phylogenetic analysis

**Figure 1:**
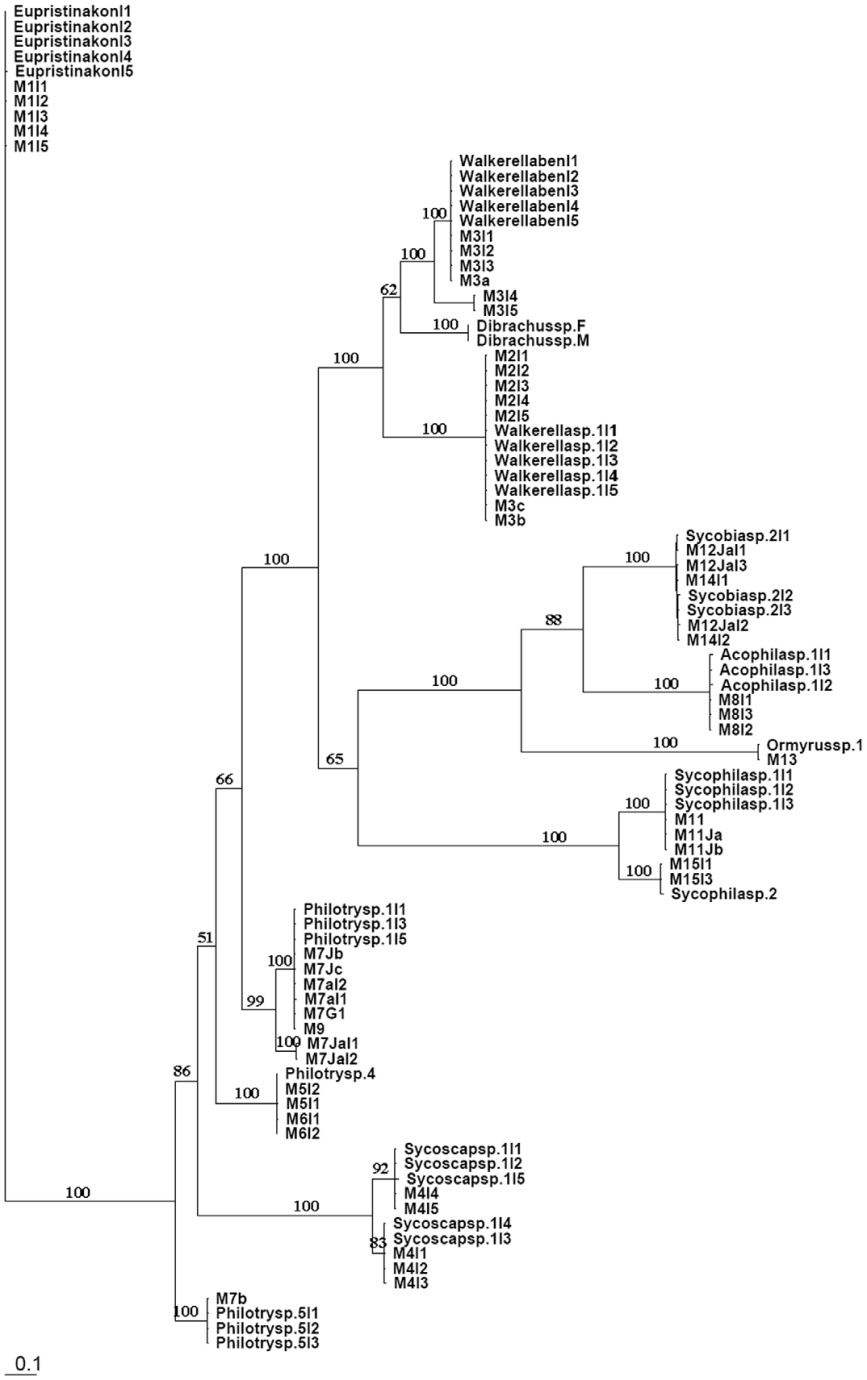
ITS2 Bayesian inference tree for fig wasps collected from *Ficus benjamina*. Values on the nodes are Bayesian posterior probabilities. The tree shows a good clustering of all the individuals from the same species, especially for polymorphic species such as *Philotrypesis* sp.1 and *Walkerella*. Cryptic species (from genera *Sycoscapter*) were also indicated.

**Figure 2:**
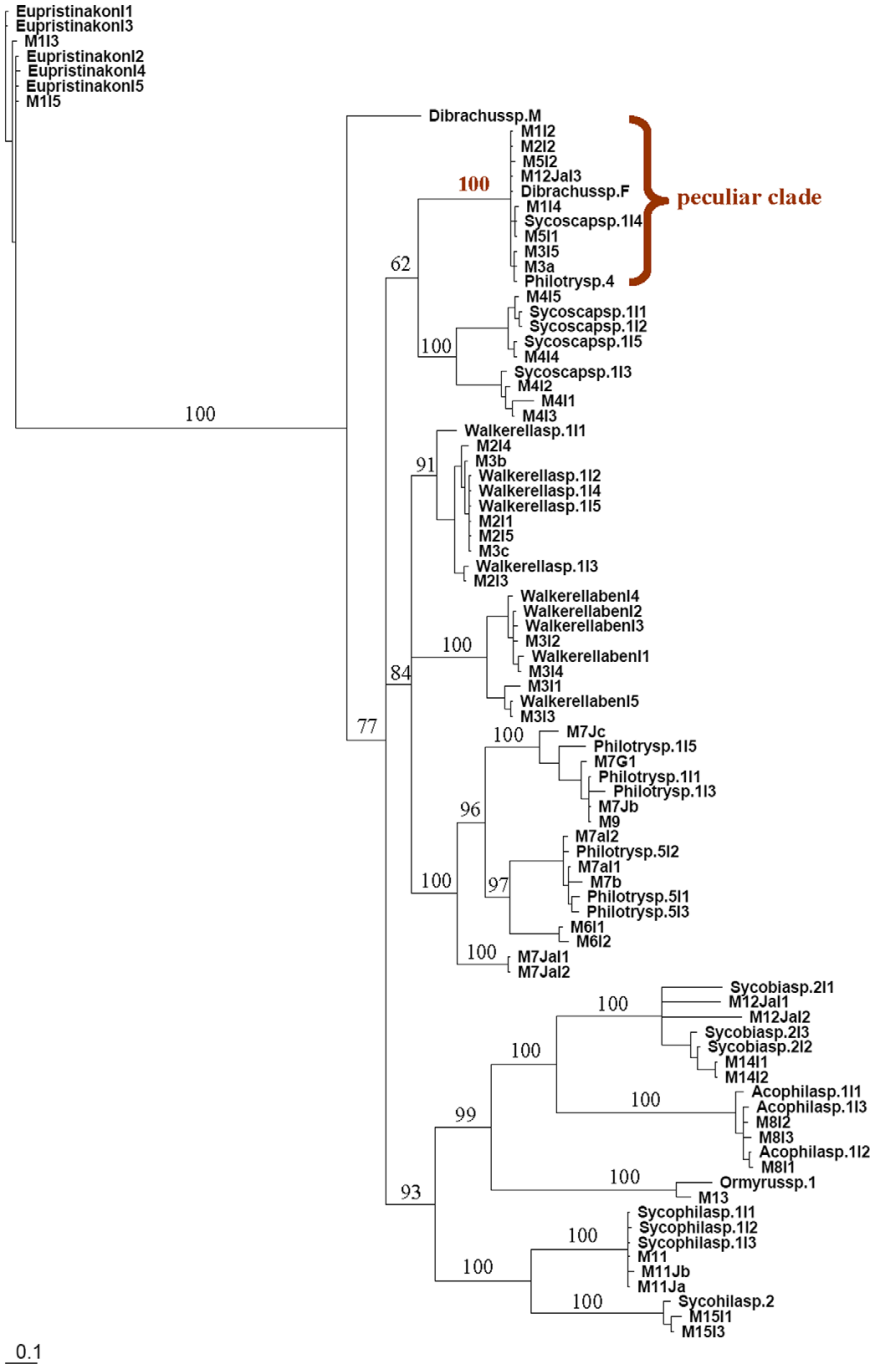
COI Bayesian inference tree for fig wasps collected from *Ficus benjamina*. Values on the nodes are Bayesian posterior probabilities. The tree shows a clustering pattern similar to that of ITS2, the only exception being a ‘peculiar clade’ with 100% support that includes 11 individuals across 8 species.

Log likelihood ratio tests indicate that GTR+I+G was the best model for all gene fragments. The Bayesian inference trees constructed from ITS2 and COI are shown in Figures 1 and 2, respectively. The trees of Cytb and EF1-α are included as supplemental figures (S2 and S3). NJ and Bayesian trees are very similar in topology and relative levels of nodal support.

We compare the molecular results with the morphospecies for the discrimination of species. When one molecular marker identifies molecular species consistent with observed morphospecies, we consider that this marker identifies the individuals into true fig wasp species. We also allow for the possibility that molecular data further split morphospecies into more cryptic species. Then all the results from the other molecular markers are tested based on the present species identification pattern.

In the Bayesian tree generated from ITS2 (Figure 1), all individuals of a morphological species—Agaonidae 1 sp.; Otitesellinae 2 sp.; Sycoryctinae 4 sp.; Epichrysomallinae 2 sp.; Eurytomidae 2 sp.; Ormyridae 1 sp.—cluster together with high support, and the sequence divergences within any one species are less than 2%. The identification results match the morphospecies very well, so ITS2 sequences provid a powerful tool for species identification, especially for highly polymorphic species. For example, female individuals of *Philotrypesis* sp.1 cluster with the five male morphs with 100% support, although leaving the species placement of one morph uncertain. Two species of *Walkerella*, represented by five male morphs, unambiguously clustered as two distinct sister lineages and with their respective male morphs. The analyses may have also identified a cryptic species. Morphological characters suggest the presence of one species of *Sycoscapter*. However, the sequence data point to the possibility of two species. DNA fragments differ in sequence length by 29 bp. Further, these two lineages cluster together as sister taxa and with high or moderate levels of support in all analyses. However, confirmation of the existence of two species should be based on additional studies.

The tree from COI (Figure 2) indicates similar sets of relationships and also with high support. However, we find one peculiar clade with 100% support that included 11 individuals from 8 species, a major deviation from the expected associations. It is very likely that this clade represents repeated amplification of a nuclear psuedogene (NUMT). The sequences in this clade show very limited sequence variation and we also have further evidence for NUMTS from the same species using different COI primer sets.

The tree from EF1-α (supplemental Figure S2) is not well resolved into species and indeed contains a large comb of 37 individuals of various species with very similar sequences. This region therefore appears unsuitable for identifying fig wasp species here.

Phylogenies derived from Cytb sequences (supplemental Figure S3) differ considerably from both ITS2 and COI trees. However, Cytb correctly assigns all individuals of some species, such as *Philotrypesis* sp.5, *Sycophila* sp.2. The 11 individuals in the peculiar clade of COI are dispersedly distributed on the Cytb tree.

## Discussion

### Combined molecular markers discriminate species

The co-existence of both cryptic and polymorphic species of fig wasps has rendered taxonomy both difficult and error-prone. This has been especially true in complex communities of wasps on monoecious figs that often contain more than 10 and even up to 30 species of insects. The assignment of male morphs to their female counterparts has proven to be a difficult task. In order to test the efficiency of molecular markers, we collected 12 morphological species of fig wasps from *Ficus benjamina*, of which six had polymorphic males and seven were extremely sexually dimorphic. We used four molecular markers to test and compare their utility in identifying fig wasp species through DNA data. From one to five specimens of each morph were used for the analysis. The four markers performed differently. In general, nucleotide sequences of COI and especially ITS2 successfully associated polymorphic individuals of a species, even when there were many different male morphs.

Many traditionally recognized morphological species are complexes of cryptic species and molecular markers can discover them via genetic distances. Fig wasps are no exception. For example, pollinating fig wasps contain cryptic species [4], [8] and it was therefore not surprising that we detected a likely cryptic species of non-pollinator fig wasp. Taken together, these examples indicate that fig wasp communities harbour many undiscovered cryptic species. The cryptic species may recognize each other using chemical or behavioural mating signals not obvious to researchers. In addition, there could also be subtle unidentified morphological traits or other features [1]. Regardless, the documentation of cryptic species of fig wasps can identify possible cases of competition and micro-ecological differentiation within the enclosed syconium space. It can also inform interesting evolutionary topics such as speciation, coevolution, sex allocation theory, and adaptation.

### Precautions for DNA barcoding fig wasps

Mitochondrial DNA has been broadly used in phylogenetic analysis. Sequencing it is often easy, it has a relatively high evolutionary rate, and it rarely recombines. One gene, COI, has been recommended for barcoding all animal species [32]. It provides excellent results in most animal groups, and gives a standard for species identification. However, the standard fragment is difficult to amplify and often generates NUMT sequences in fig wasps, so further exploration of markers is desirable. Here, ITS2 is revealed to be an excellent molecular marker for identifying species of fig wasps, while COI yields some problematic results. One clade in the COI tree contains 11 individuals from several genera and this association strongly deviates from the expectations. Whereas sequence divergences between groups normally ranges from 11.1% to 22.0%, that within the unexpected clade is less than 0.5%, and more akin to that within species. This most likely represents a clade of NUMT sequences and the corresponding individuals appear in many different clades in the tree constructed from Cytb sequences. Whereas ITS2 occurs in the nuclear genome, both COI and Cytb are mtDNA genes that may be transposed to the nuclesr genome as extra copies that can confuse phylogenetic analyses.

NUMTs evolve much more slowly than their mitochondrial homologues. When used in phylogenetic analysis, they can yield an unusual tree topology because they can form a distinct cluster and, therefore, confound the phylogenetic results [33]. Unlike *Drosophila melanogaster*, the assembled genome of *Apis mellifera* has a high density of NUMTs, even more so than in humans. Further, different regions of the mitochondrial genome have a different frequency of transferred NUMTs [34]. In fig wasps, the pattern of NUMTs is complex (Unpublished data) and some species appear to have NUMTs for COI or Cytb. NUMT sequences are often, but not always, characterized by internal stop codons and these occur in the conceptual translation of Cytb sequences in some individuals in this study (M3a, *Philotrypesis* sp.1 I5, M7JaI2, and M7Jc). Thus, NUMTs complicate the use of mtDNA in identifying fig wasp species in some cases.

A related issue is that symbiotic *Wolbachia* bacteria can drive the evolution of different mtDNA clades within a single species that based on mtDNA data alone might be considered different species [e.g. Delgado & Cook 2009. BMC Evol. Biol] [12], [13]. Infections of *Wolbachia* can sweep through host populations, removing most or all mtDNA variation and establishing the haplotype linked to the infection as dominant. If hybridization occurs, *Wolbachia* could cause similar sweeps in two species, confounding the use of mtDNA alone to identify species [35], [36]. Fig wasps have a very high incidence of *Wolbachia* infection with about 70% of species infected [4], [37], [38]. Consequently, *Wolbachia* might have a role in explaining the mtDNA phylogenies observed here though NUMTS seem a more likely explanation for the major discrepancies.

In our study, because mtDNA markers information is difficult to interpret, a new marker may be desirable and nuclear ITS2 is clearly a very good candidate. It my also be useful in other taxa and may often be a strong complement to mtDNA data that can help identify species and check for possible NUMT or symbiont-induced complexities in the data, as these will not affect this nuclear region. However, if ITS2 is used alone, it defeats a major advantage of DNA barcoding, i.e. the use of one gene fragment for identifying species all animal taxa. For this reason, combination of ITS2 with COI may be a good approach as it allows both connectability of data sets with quality control for complicating issues. ITS2 is not a good general alternative to COI because it also has problems. It is a noncoding region that evolves fast and makes alignment and analysis difficult beyond quite small taxonomic distances. In addition, it is a multi-copy fragment and some species show variation between different copies in the same species. There is probably no universal panacea but combining more general mtDNA approaches with a more locally defined nuclear marker is a good option.

Misleading barcodes can be discovered by using gene fragments from both the mtDNA and nuDNA genomes. Accuracy may require multiple markers. Here, conflict is useful because it highlights potential issues with NUMTs and/or *Wolbachia*. The necessity of using multiple genes might be detected after attempting to translate coding genes and performing BLAST searches in GenBank.

## Supporting Information

Figure S1 Morphological images of fig wasps collected from *Ficus benjamina*. Names of species for all images, including both females and males, correspond to those in the phylogenetic trees (Figures 1, 2, Supplemental Figure S2, and Supplemental Figure S3).
(PDF)

Figure S2 Figure legend: EF1-α Bayesian inference tree for fig wasps collected from *Ficus benjamina*. It appears as a large brush comprised of 37 individuals of various species. These sequences did not correctly identify species.
(TIF)

Figure S3 Figure legends: Cytb Bayesian inference tree for fig wasps collected from *Ficus benjamina*. Values on the nodes are Bayesian posterior probabilities. This tree shows a different clustering pattern compared with ITS2 and COI, and the relationships of many individuals remain unresolved. The marked taxa indicate all individuals in the ‘peculiar clade’ in COI tree (Figure 2).
(TIF)

Table S1 This table provides morphological diagnoses for all the female species and male morphs presented in Supplemental Figure S1. (Note: The species identity of some unresolved morphs remains elusive. For example, the two individuals of M7Ja are not classified as either *Philotrypesis* sp.1 or *Philotrypesis* sp.5. Analyses indicate the need for extended sampling and mating evidence.)
(DOC)
