# Supplementary figures and images for: Molecular Approaches to Identify Cryptic Species and Polymorphic Species within a Complex Community of Fig Wasps
Source: PLoS One. 2010 Nov 29;5(11):e15067. doi: 10.1371/journal.pone.0015067 (PMC2993961; doi:10.1371/journal.pone.0015067)

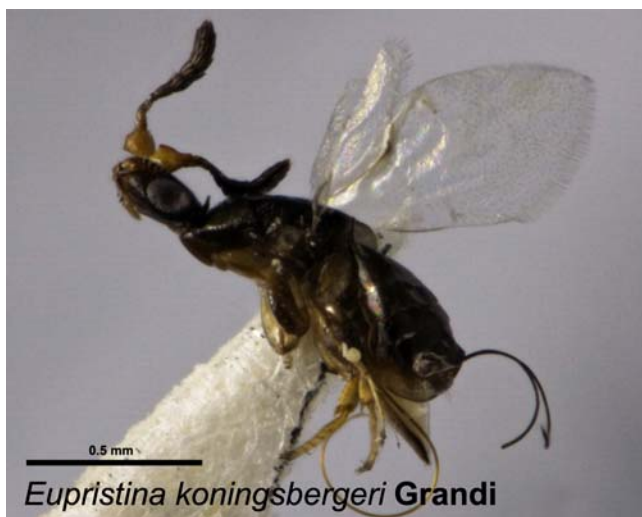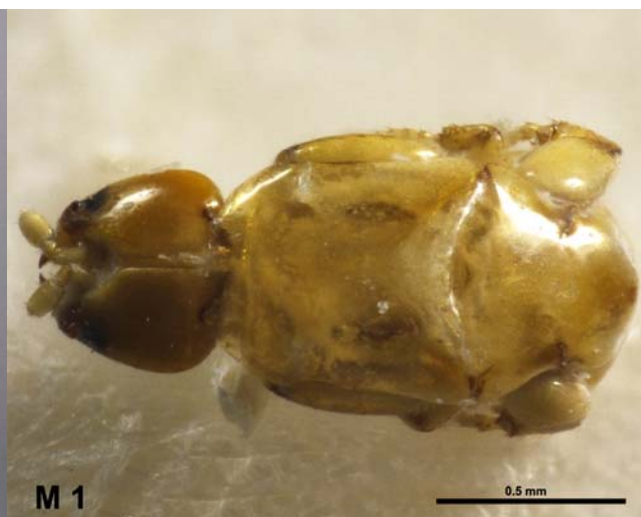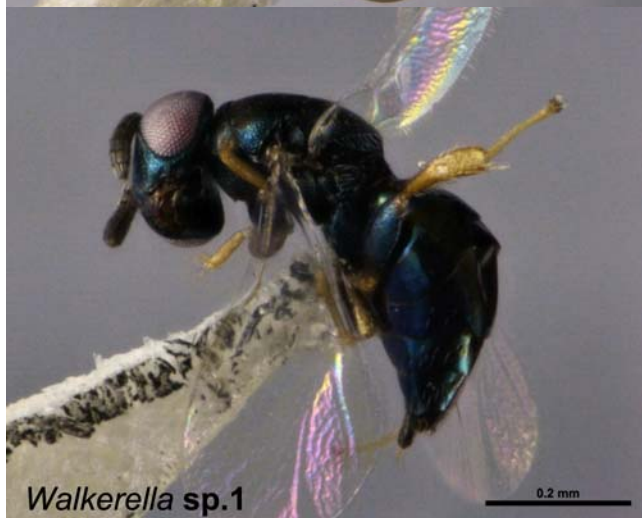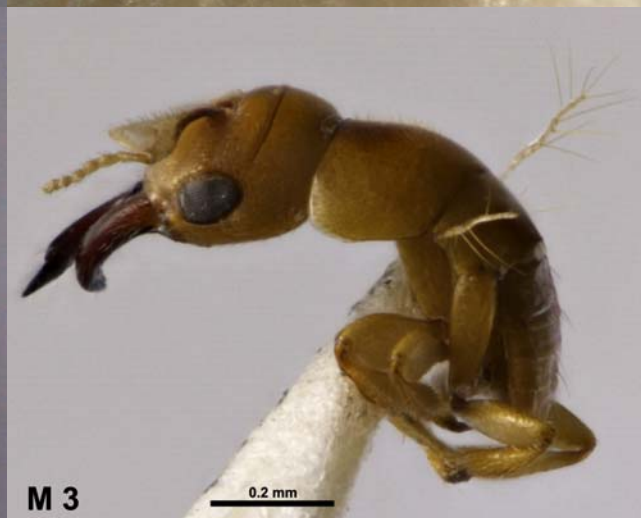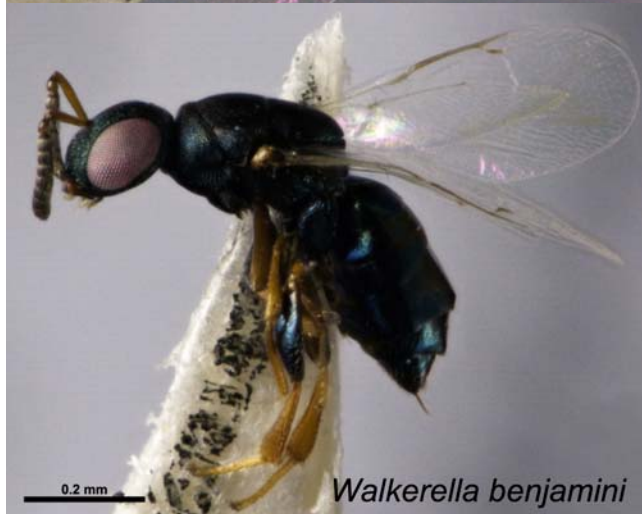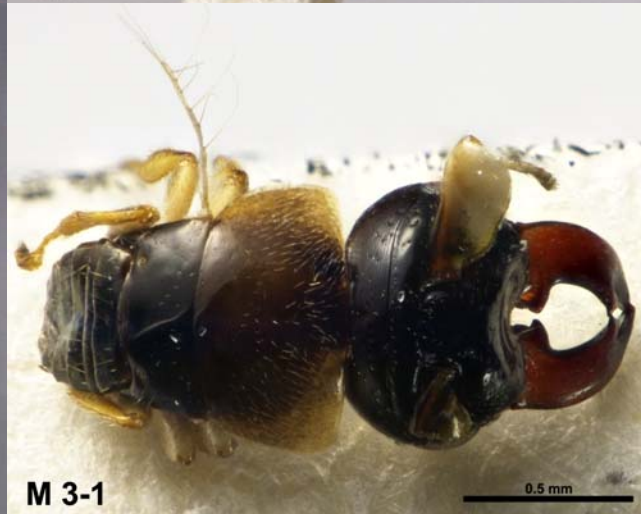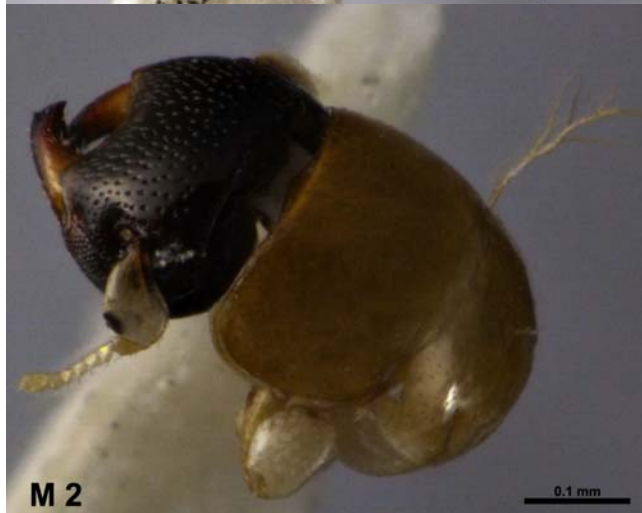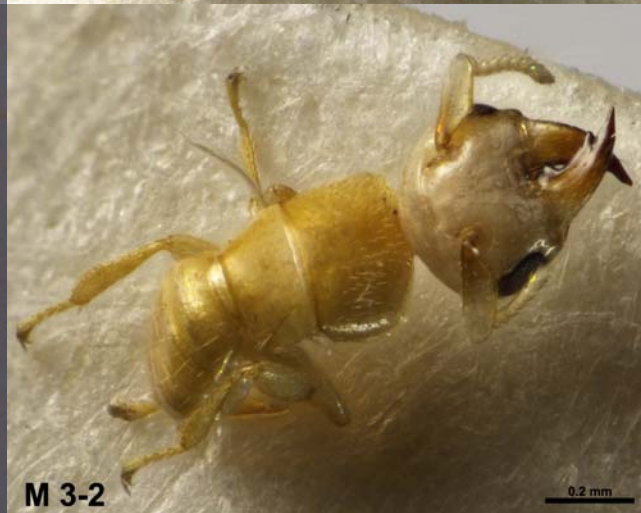

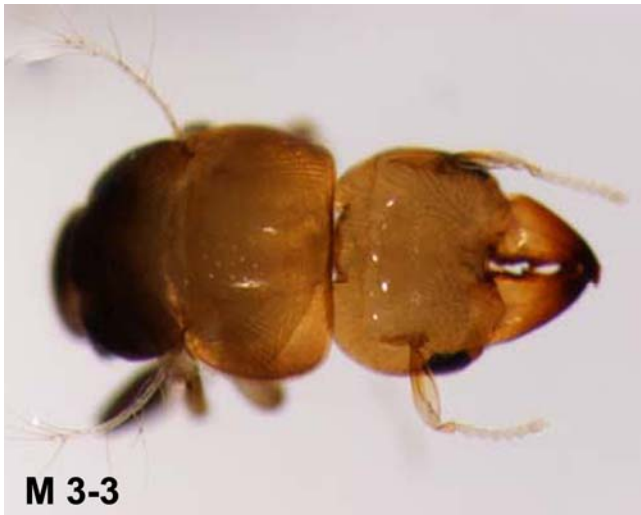

**M 3-3**

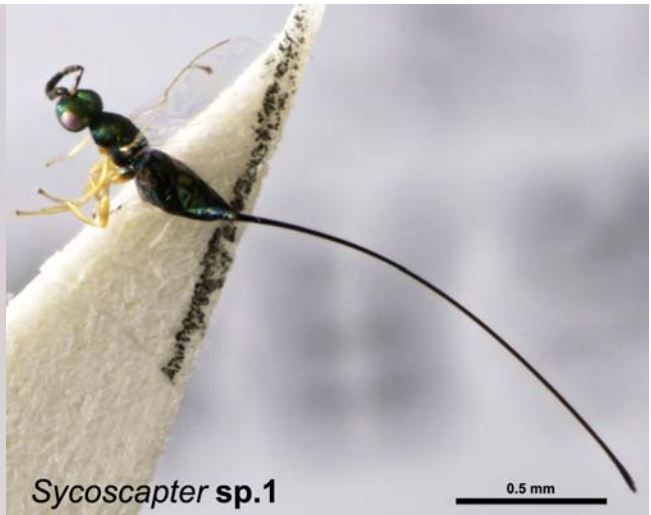

*Sycoscapter* sp.1

0.5 mm

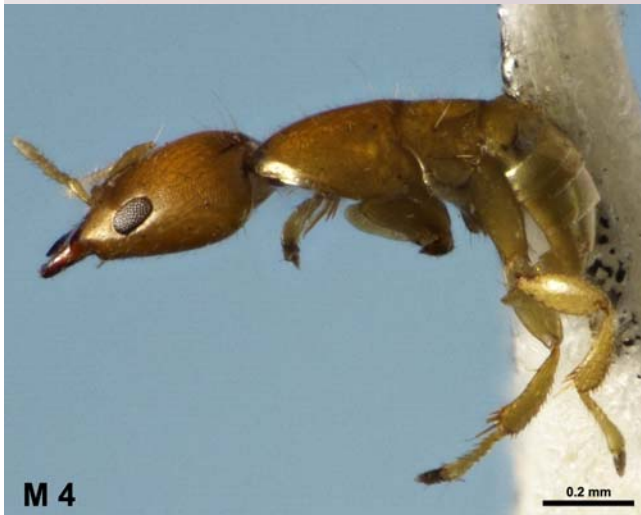

**M 4**

0.2 mm

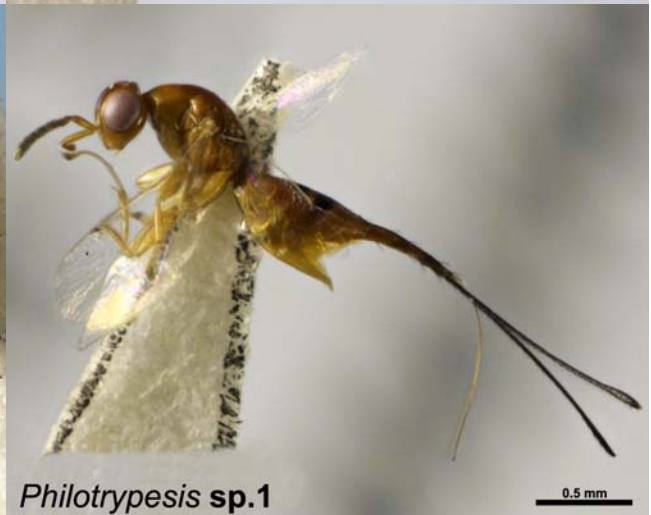

*Philotrypesis* sp.1

0.5 mm

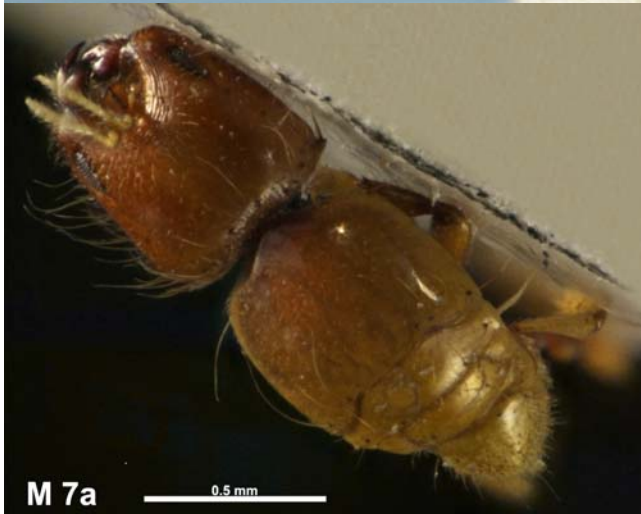

**M 7a**

0.5 mm

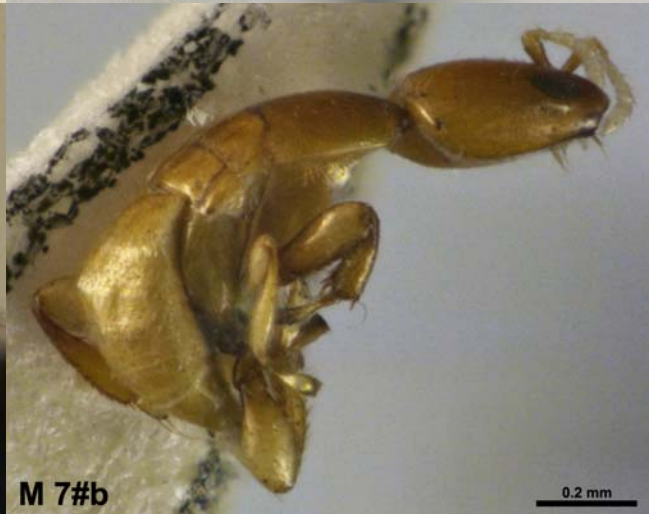

**M 7#b**

0.2 mm

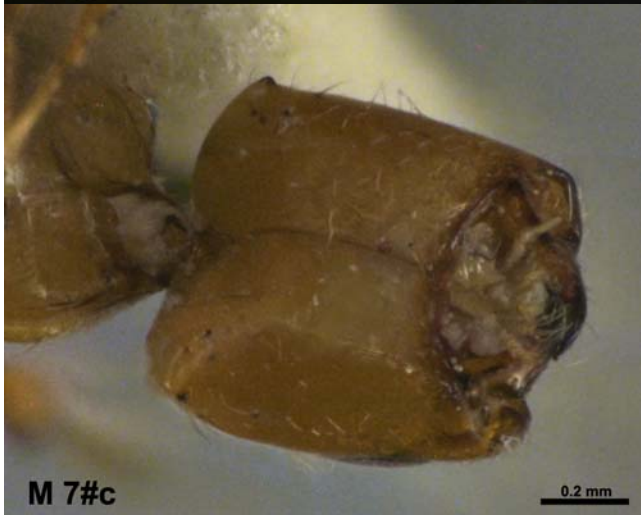

**M 7#c**

0.2 mm

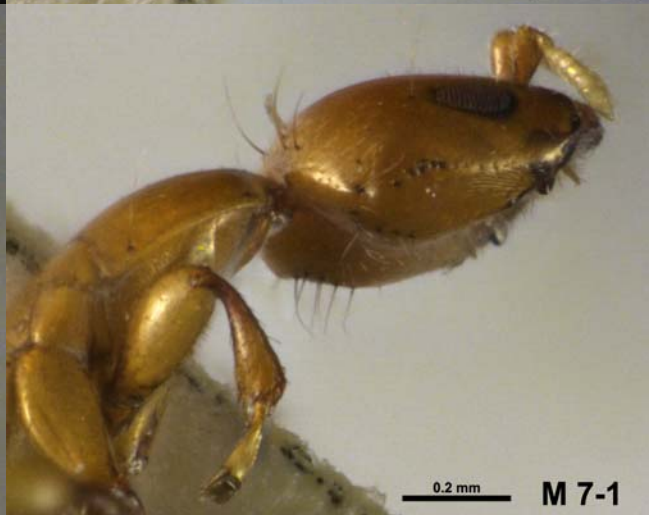

**M 7-1**

0.2 mm

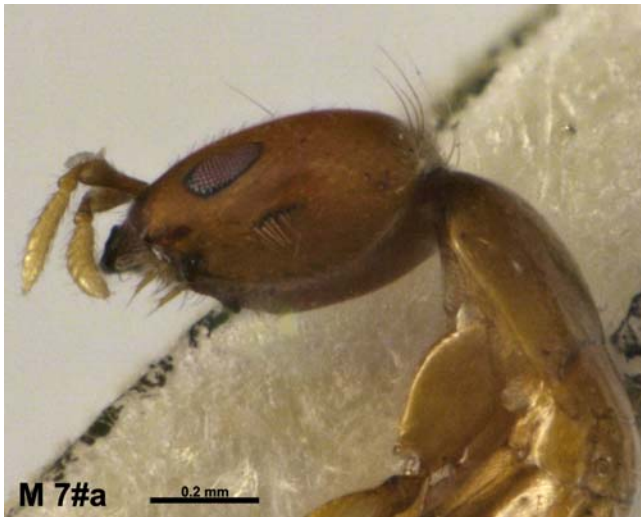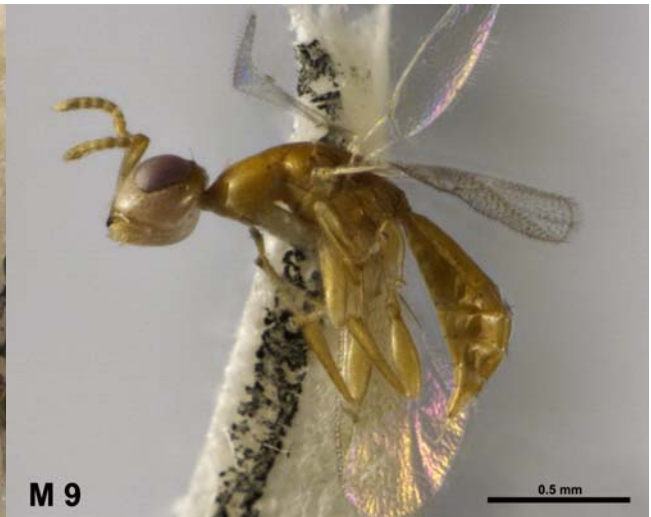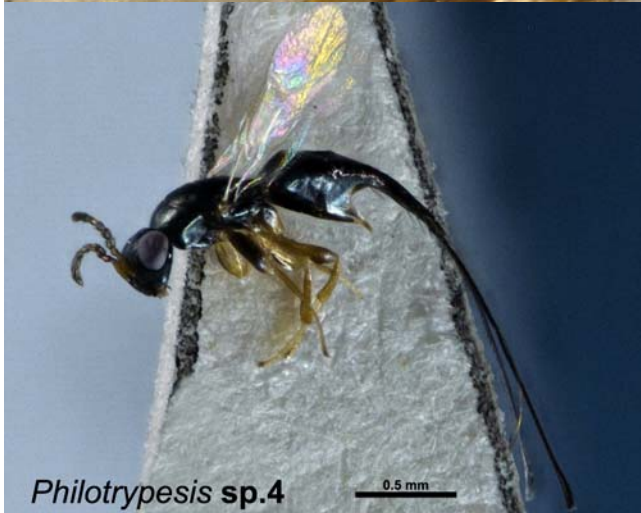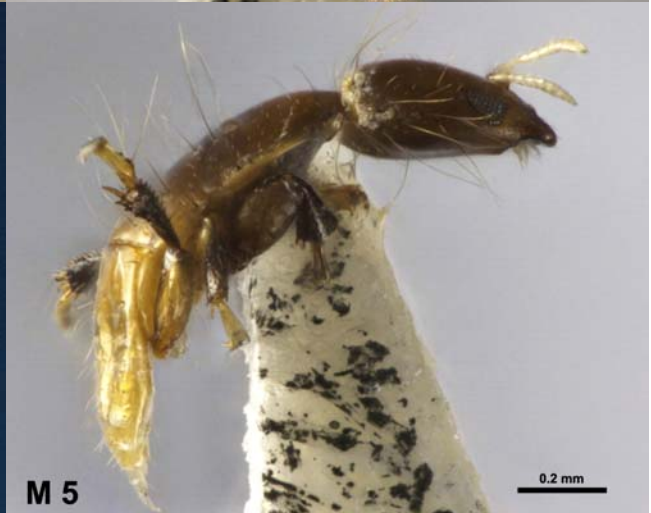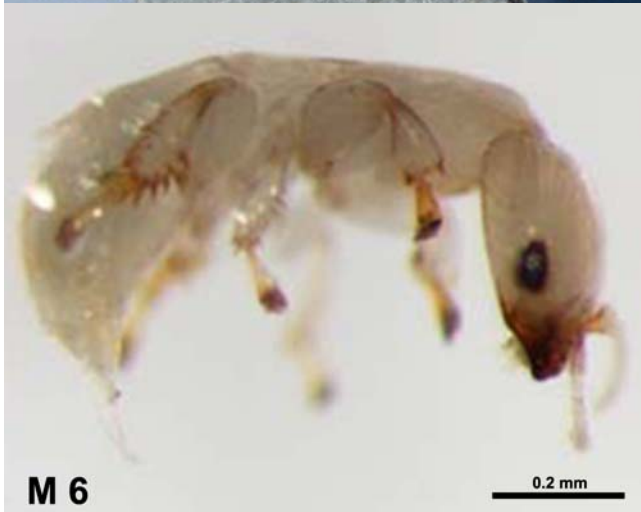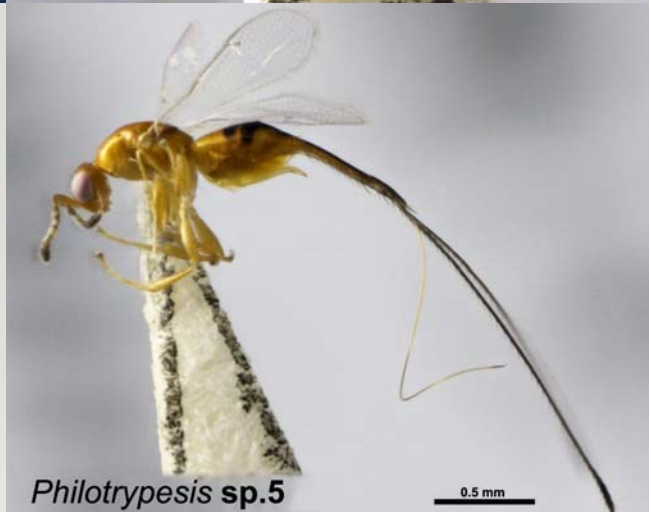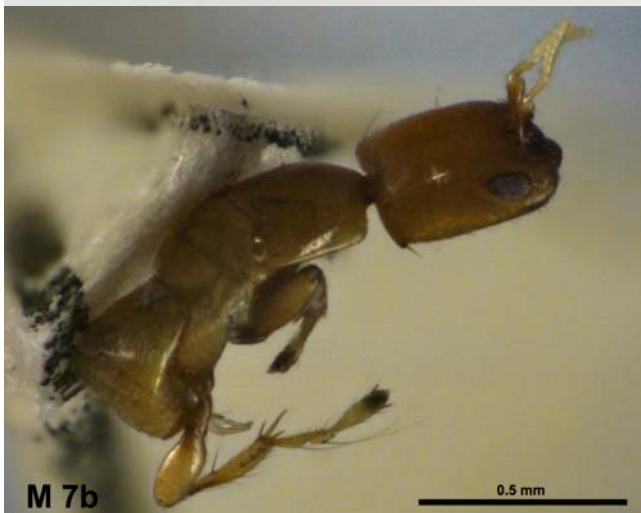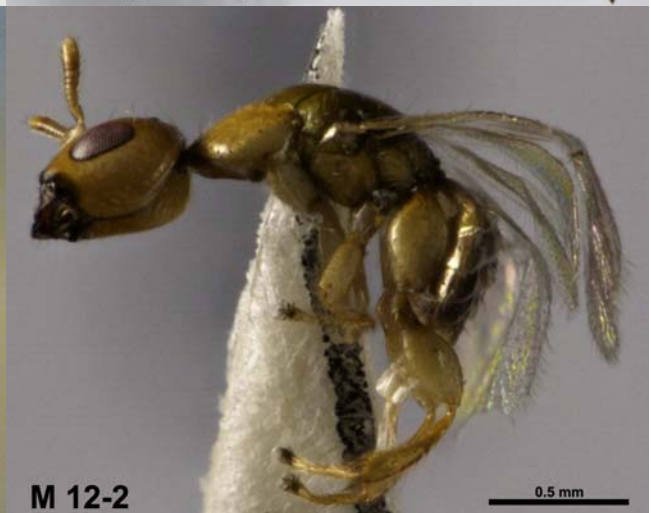

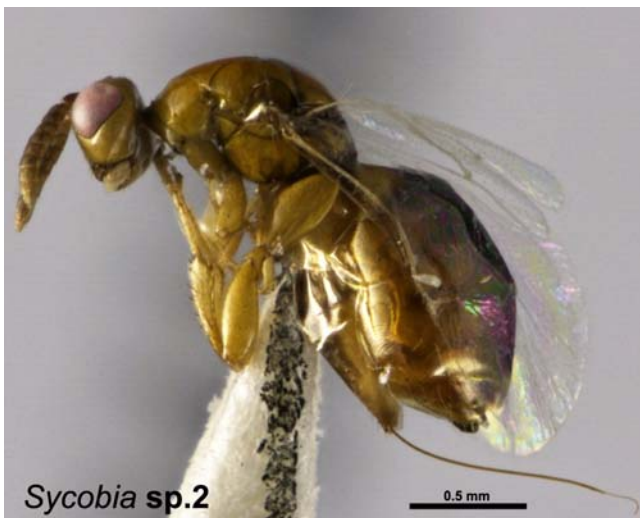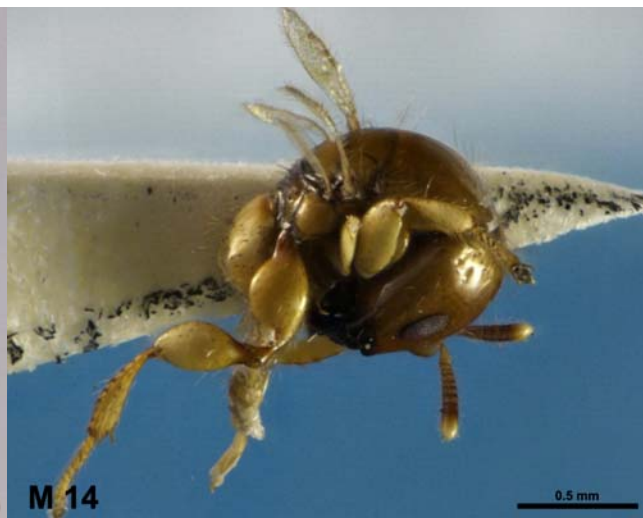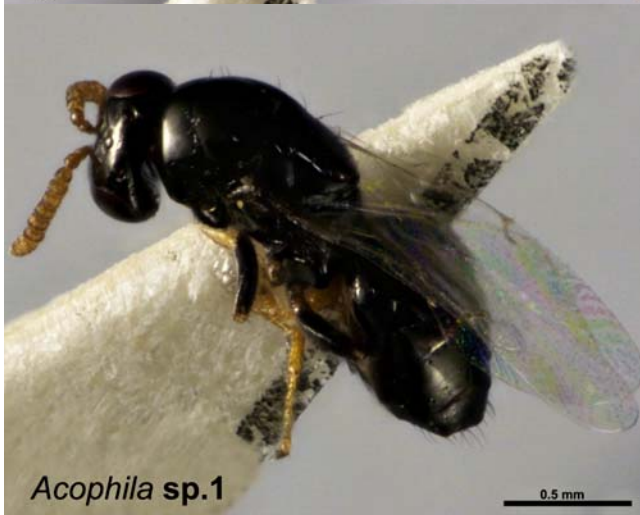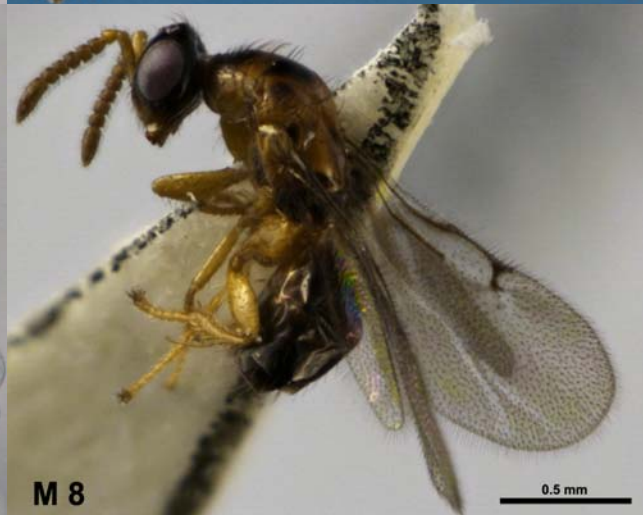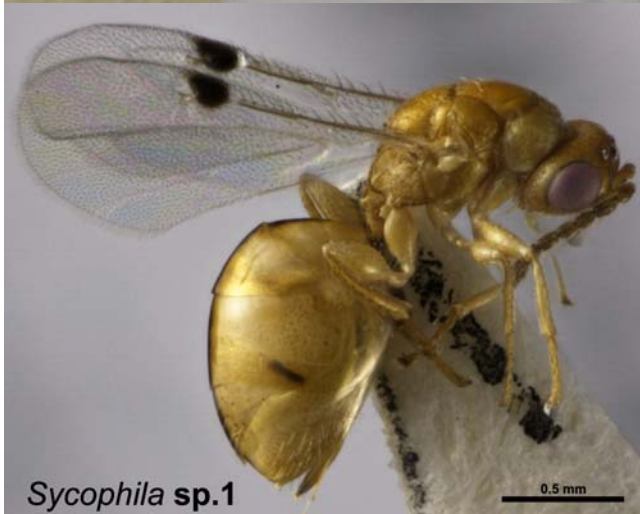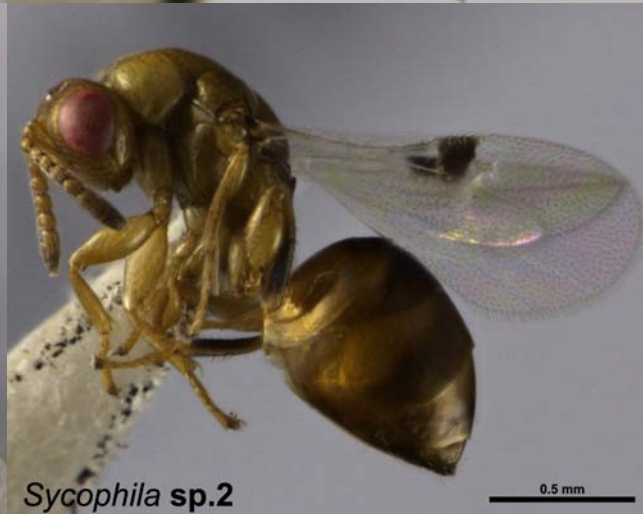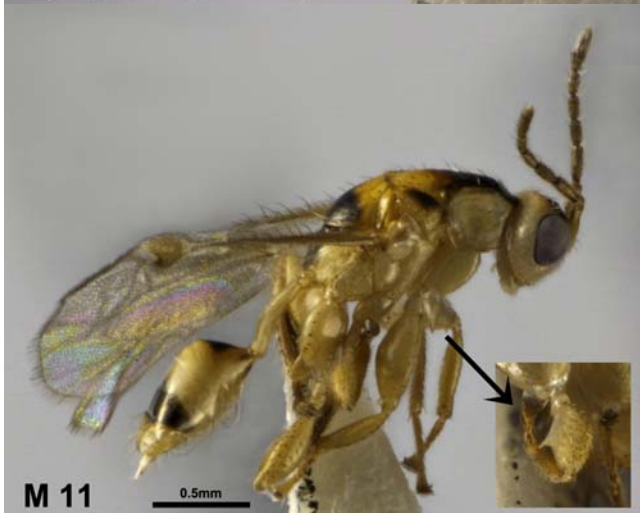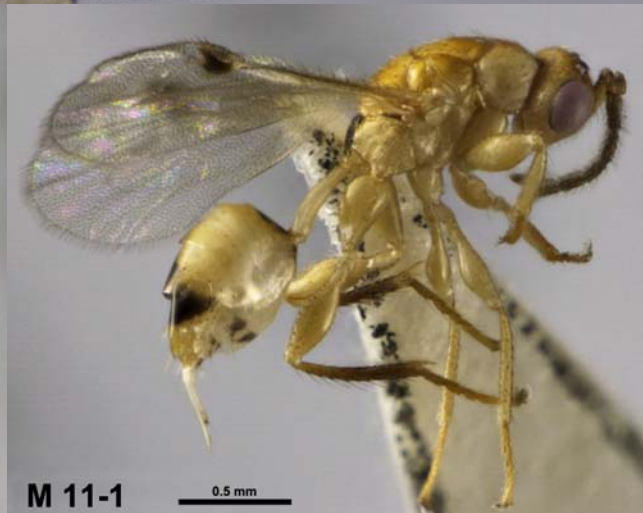

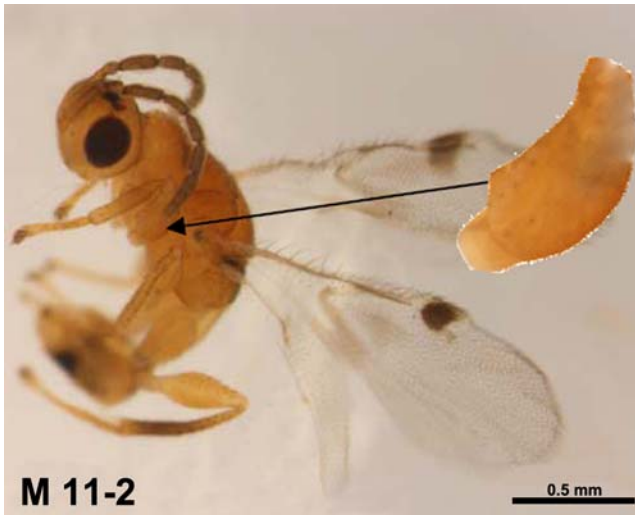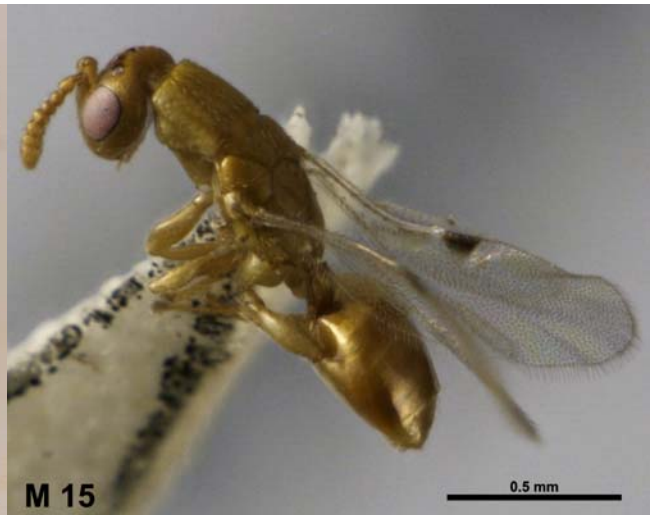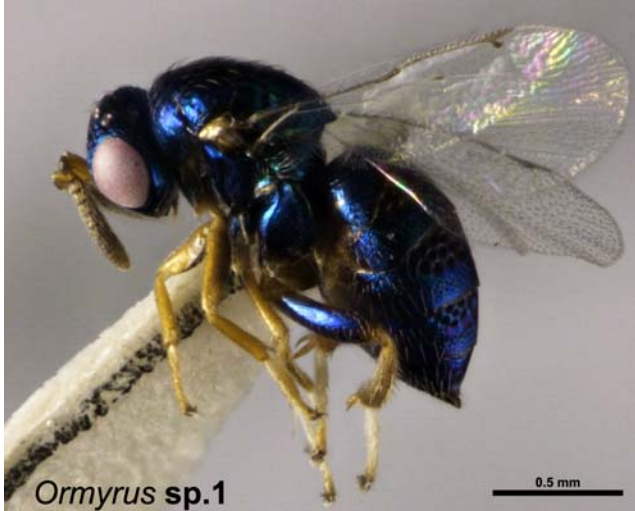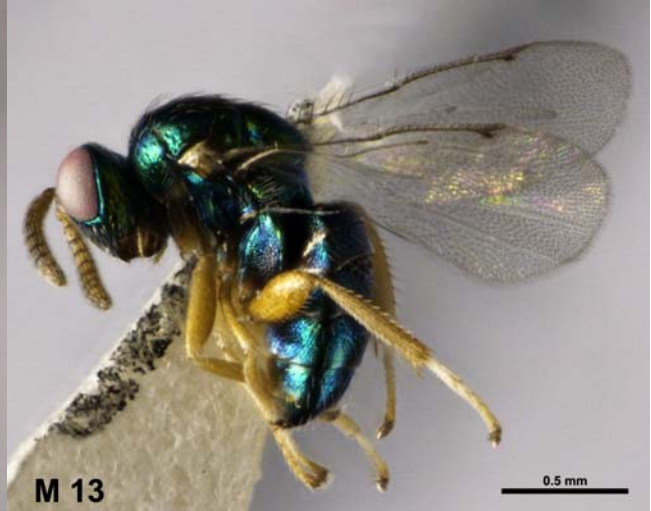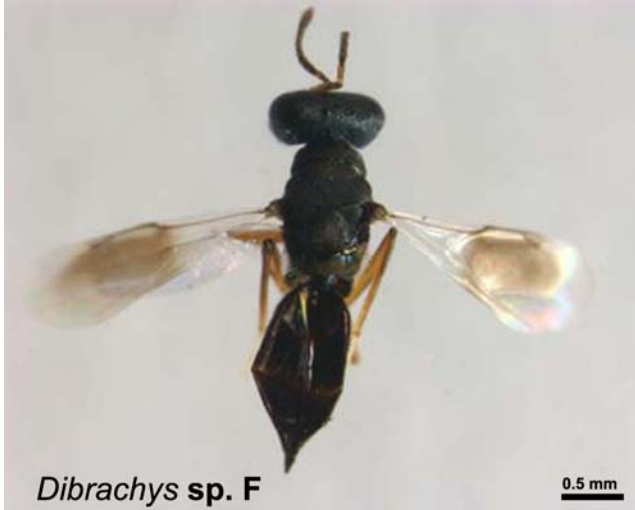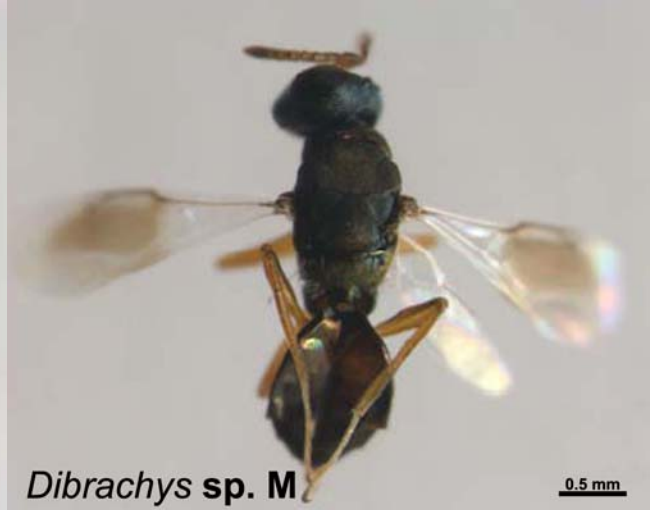

Supplement: Figure S1 — Morphological images of fig wasps collected from Ficus benjamina. Names of species for all images, including both females and males, correspond to those in the phylogenetic trees (Figures 1, 2, Supplemental Figure S2, and Supplemental Figure S3). (PDF) [file pone.0015067.s001.pdf]

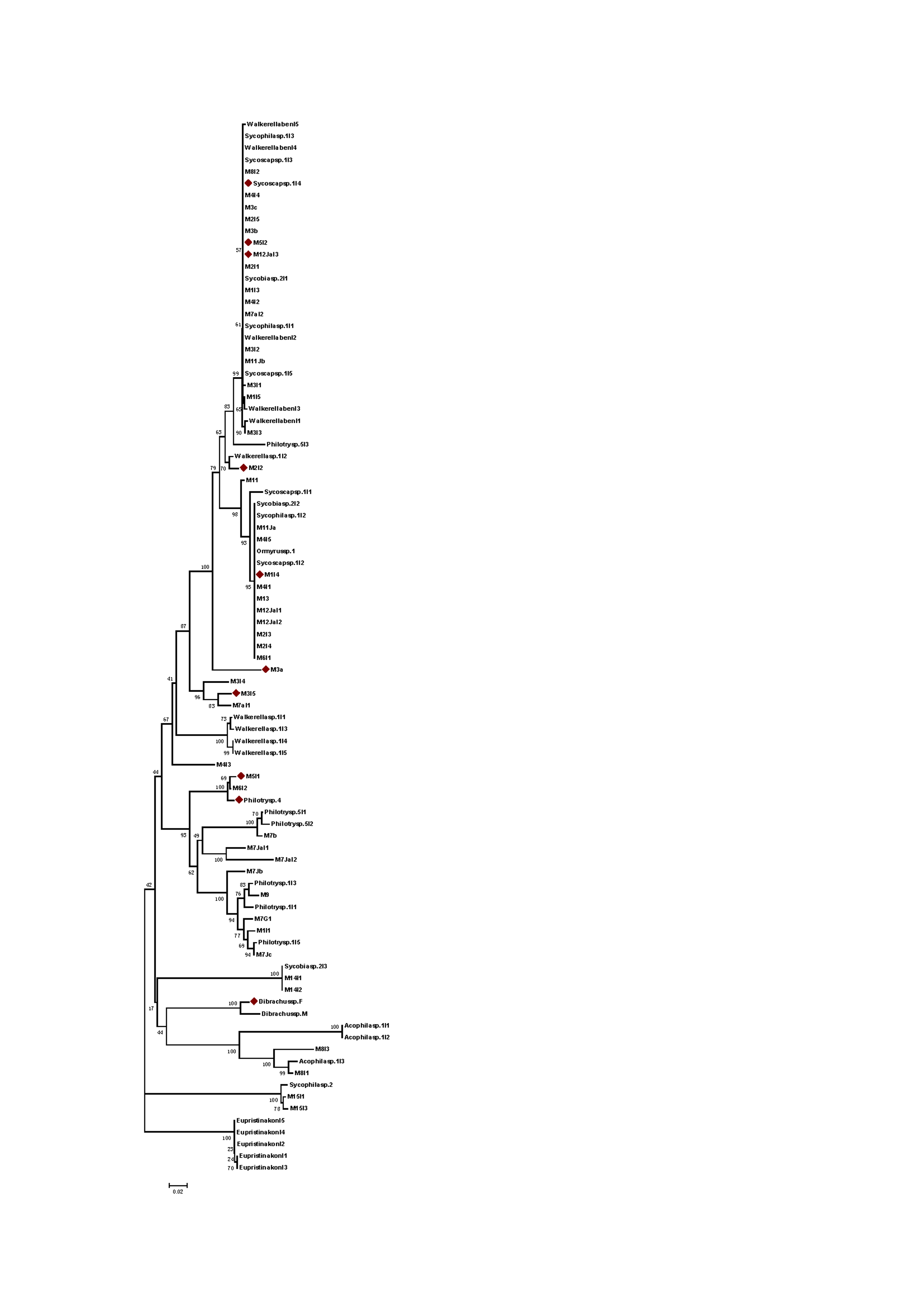

Supplement: Figure S3 — Figure legends: Cytb Bayesian inference tree for fig wasps collected from Ficus benjamina. Values on the nodes are Bayesian posterior probabilities. This tree shows a different clustering pattern compared with ITS2 and COI, and the relationships of many individuals remain unresolved. The marked taxa indicate all individuals in the ‘peculiar clade’ in COI tree (Figure 2). (TIF) [file pone.0015067.s003.tif]
