# Supplementary material for: Molecular Approaches to Identify Cryptic Species and Polymorphic Species within a Complex Community of Fig Wasps
Source: PLoS One. 2010 Nov 29;5(11):e15067. doi: 10.1371/journal.pone.0015067 (PMC2993961; doi:10.1371/journal.pone.0015067)
Supplement: Table S1 — This table provides morphological diagnoses for all the female species and male morphs presented in Supplemental Figure S1. (Note: The species identity of some unresolved morphs remains elusive. For example, the two individuals of M7Ja are not classified as either Philotrypesis sp.1 or Philotrypesis sp.5. Analyses indicate the need for extended sampling and mating evidence.) (DOC) [file pone.0015067.s004.doc]

Table S1. Samples of Females and Male Morphs and Their Morphological Diagnostics

| Family and Subfamily | Species and Sex | Species code | Sampled individuals | Diagnostics Characters |
| --- | --- | --- | --- | --- |
| Agaonidae  Agaoninae | *Eupristina koningsbergeri* Grandi | Eupristinakon | 5 | Eye two times as long as malar sulcus; appendage of 3rd antennal segment long and spinal; gastral spiracle oval. |
|  | M1 | M1 | 5 | Wingless; antennae situated in a common scrobal cavity; anellus distinct; propodeum less than 2 times as wide as long; spiracle situated in anterior propodeum; hind tarsi five-segmented. |
| Pteromalidae  Otitesellinae | *Walkerella benjamini* (Joseph, 1957) | Walkerellaben | 5 | Ovipositor tip straight, not protruding, or a little beyond gaster; clypeal margin medially emarginated. |
|  | M3 | M3 | 5 | With vestigial wings; head yellow; mandibles strong, more than half of head length, with inner edge extremely curved, forming a big cavum between two mandibles. |
|  | M3-1a | M3a | 1 | With vestigial wings; separated from M3 by brown body. |
|  | *Walkerella* sp. 1 | Walkerellasp1 | 5 | Can be separated from *Walkerella benjamini* by ovipositor tip strongly bending downwards, protruding beyond gaster tip. |
|  | M2 | M2 | 5 | With vestigial wings; head black; mandibles strong, about half of head length, with inner edge curved, forming a cavum between two mandibles. |
|  | M3-2 | M3b | 1 | With vestigial wings; separated from M3-3 by smaller, yellow-white body. |
|  | M3-3 | M3c | 1 | With vestigial wings; mandible strong, about half of head length, inner edge with miniteeth, straight; hind legs black or brown, with coxae and femur swollen. |
| Pteromalidae  Sycoryctinae | *Sycoscapter* sp.1 | Syoscapsp.1 | 5 | Body green, with long ovipositor. |
|  | M4 | M4 | 5 | Wingless; antennae clavate; fore tibia with long spines, expanded towards end, fore coxae with sharp edge ventrally; oral margin not concave on ventral head. |
|  | *Philotrypesis* sp.1 | Philotrysp.1 | 3 | Body yellow, relatively large species; extended last two tergites shorter than gaster; ovipositor shorter than, or as long as, body length. |
|  | M7a | M7a | 2 | Winless; antennae slender, not clavate; head nearly quadrate, having an incision near hind corner on which a long spine; head with long hairs both dorsally and ventrally; mandible with two teeth; pronotum ventrally with a long hair on side; fore coxae with hairs inter-ventrally. |
|  | M7#b | M7Jb | 1 | Winless; head small, mandible with three teeth; fore coxae bare inner-ventrally; head without a band of bristles on sides, without a bush of bristles; ventrally near hind corner. |
|  | M7#c | M7Jc | 1 | Winless; separated from M7#b by Head relatively larger, with a bush of bristles ventrally near hind corner. |
|  | M7-1 | M7G1 | 1 | Winless; separated from M7a by head larger, with a bush of bristles ventrally near hind corner; and fore coxae bare inner-ventrally; mandibles with tree teeth. |
|  | M9 | M9 | 1 | Winged; body yellow; wings transparent; fist gastral tergite about one third of gaster length. |
|  | M7#a | M7Ja | 2 | Winless; separated from M7a by fore coxae bare, hind out corner of head rounded; head relatively larger, with a line of hairs on sides. |
|  | *Philotrypesis* sp.4 | Philotrysp.4 | 4 | Body black, relatively large species; extended last two tergites shorter than gaster; ovipositor as long as body length. |
|  | M5 | M5 | 2 | Wingless; body brown; head with long hairs which longer than head breadth; fore and hind femurs swollen. |
|  | M6 | M6 | 2 | Wingless; body tiny, white; head hairless. |
|  | *Philotrypesis* sp.5 | Philotrysp.5 | 3 | Separated from *Philotrypesis* sp. 1 by extended gastral tergites as long as gaster; ovipositor longer than body length; ovipositor sheaths are almost always loose, separated. |
|  | M7b | M7Jb | 1 | Wingless; head small, mandible with three teeth; fore coxae bare inner-ventrally; head without a band of bristles on sides, without a bush of bristles ventrally near hind corner. |
| Family (?)  Epichrysomallinae | *Sycobia* sp.2 | Sycobiasp.2 | 3 | Body yellow with stripes on the gaster; dorsum of thorax smooth. |
|  | M12-1 | M12Ja | 3 | Winged; head little broader than thorax. |
|  | M14 | M14 | 2 | With vestigial wings; head larger, much broader than thorax; notauli divergent backwards; pronotum not visible dorsally; lower face edge black. |
|  | *Acophila* sp.1 | Acophilasp.1 | 3 | Body black, smooth; occipital carina present; scutellum broadly rounded posteriorly; antennae orange. |
|  | M8 | M8 | 3 | Winged; body smooth, hairy; body coloration varying from brown with black maculae to completely black; fore wing hairy, with a transparent narrow band along cubital vein. |
| Eurytomidae | *Sycophila* sp.1 | Sycophilasp.1 | 3 | Body yellow; scutellum smooth; eyes brown; hind tibia yellow; gaster yellow with black transverse stripes; propodeum with different sculptures. |
|  | M11 | M11 | 1 | Winged; fore femur with sharp tooth on front; dorsum of mesosoma yellow with black marks. |
|  | M11-1 | M11Ja | 1 | Winged; separated from M11 by fore femur without sharp tooth on front; dorsum of mesosoma yellow without black marks, except median area of propodeum; usually smaller than M11. |
|  | M11-2 | M11Jb | 1 | Winged; separated from M11 by fore femur with blunt tooth on front; dorsum of mesosoma yellow with less or smaller black marks. |
|  | *Sycophila* sp.2 | Sycohilasp.2 | 1 | Separated from *Sycophila* sp. 1 by scutellum with coarse round sculpture; eyes red; gastral petiole black or brown; gaster black or brown; Hind tibia black or brown; propodeum with anterior 1/3 black or brown. |
|  | M15 | M15 | 2 | Winged; antennae stout; pronotum elongate; with long gastral petiole. |
| Ormyridae | *Ormyrus* sp.1 | Ormyrussp.1 | 1 | Winged; gaster with coarse sculpture; marginal vein more than 7 times as long as short stigmal vein; antenna formula 11263, funicular segments transverse. |
|  | M13 | M13 | 1 | Winged; gastral sculpture fine; marginal vein long; first funicular segments quadrate. |
| Pteromalidae | *Dibrachys* sp. F | Dibrachussp.F | 1 | Winged; normal pteromalid female; fig unassociated. |
|  | *Dibrachys* sp. M | Dibrachussp.M | 1 | Winged; normal pteromalid male. |
